# METTL3-dependent MALAT1 delocalization drives c-Myc induction in thymic epithelial tumors

**DOI:** 10.1186/s13148-021-01159-6

**Published:** 2021-09-16

**Authors:** Alessia Iaiza, Claudia Tito, Zaira Ianniello, Federica Ganci, Valentina Laquintana, Enzo Gallo, Andrea Sacconi, Silvia Masciarelli, Luciana De Angelis, Sara Aversa, Daniele Diso, Marco Anile, Vincenzo Petrozza, Francesco Facciolo, Enrico Melis, Edoardo Pescarmona, Federico Venuta, Mirella Marino, Giovanni Blandino, Giulia Fontemaggi, Alessandro Fatica, Francesco Fazi

**Affiliations:** 1 Department of Anatomical, Histological, Forensic and Orthopedic Sciences, Section of Histology and Medical Embryology, Sapienza University of Rome, Laboratory Affiliated to Istituto Pasteur Italia-Fondazione Cenci Bolognetti, Via A. Scarpa, 14-16, 00161 Rome, Italy; 2 Department of Biology and Biotechnology ‘Charles Darwin’, Sapienza University of Rome, P.le Aldo Moro, 5, 00185 Rome, Italy; 3 Oncogenomic and Epigenetic Unit, IRCCS Regina Elena National Cancer Institute - IFO, Rome, Italy; 4 Department of Pathology, IRCCS Regina Elena National Cancer Institute, Rome, Rome, Italy; 5 Histology and Embryology Section, Department of Life Science and Public Health, Fondazione Policlinico Universitario A. Gemelli IRCCS, 00168 Rome, Italy; 6 Pathology Unit, ICOT, Department of Medico-Surgical Sciences and Biotechnologies, Sapienza University of Rome, Latina, Italy; 7 Department of Thoracic Surgery, Sapienza University of Rome, Rome, Italy; 8 Thoracic Surgery, IRCCS Regina Elena National Cancer Institute - IFO, Rome, Italy

**Keywords:** m^6^A, METTL3, c-MYC, MALAT1, Thymic epithelial tumors, Thymic carcinoma, lncRNAs, JQ1 inhibitor, S6K1

## Abstract

**Background:**

Thymic epithelial tumors (TETs) are rare neoplasms, originating from epithelial thymic cells. The oncogenic potential of these rare neoplasms is still largely undefined, and a deeper molecular characterization could result in a relevant advance in their management, greatly improving diagnosis, prognosis and treatment choice. Deregulation of N6-methyladenosine (m^6^A) RNA modification, catalyzed by the METTL3/METTL14 methyltransferase complex, is emerging as a relevant event in cell differentiation and carcinogenesis. Various studies have reported that altered expression of METTL3 is associated with an aggressive malignant phenotype and favors migration and invasiveness, but its role in Thymic Tumors remains unknown.

**Results:**

In this study, we characterized that METTL3 contributes to Thymic Epithelial Tumor phenotype. We evidenced that METTL3 is overexpressed in tumor tissue compared to normal counterpart. Silencing of METTL3 expression in thymic carcinoma cells results in reduced cell proliferation and overall translation rate. Of note, METTL3 is responsible for the induction of c-MYC expression in TET cells. Specifically, high expression of c-MYC protein is enabled by lncRNA MALAT1, which is methylated and delocalized by METTL3. Interestingly, blocking of c-MYC by using JQ1 inhibitor cooperates with METTL3 depletion in the inhibition of proliferation and induction of cell death.

**Conclusion:**

This study highlighted METTL3 as a tumor promoter in Thymic tumors and c-MYC as a promising target to be exploited for the treatment of TET.

**Supplementary Information:**

The online version contains supplementary material available at 10.1186/s13148-021-01159-6.

## Background

Thymic epithelial tumors (TETs) are very rare neoplasms comprising thymoma (THY) and thymic carcinoma (TC) that establish a unique group of cancers arising from the epithelial cell of the Thymus [1]. On the basis of the current World Health Organization (WHO) histopathological classification, thymomas have been classified into types A, AB, B1, B2, and B3 and rare other subtypes [2, 3].

The malignant potential of thymomas, ranging from low to very aggressive locally, is in relation to the stage of these tumors [4, 5]. The metastatic potential and local relapse and/or pleural dissemination that characterize both the advanced stage of B2 and B3 types of thymomas and thymic carcinomas (TC) is similar to the other carcinoma features observable outside of mediastinum [6].

Complete surgical resection is the gold standard when feasible; chemotherapy like the combination of cisplatin, doxorubicin and cyclophosphamide (PAC) and/or radiotherapy can be administered as an induction at advanced stages or prior to surgery as adjuvant treatment in case of advanced pathological stages [7, 8].

The management of TET is a paradigm of synergistic strategy to guarantee an advance in clinical and translational research in these tumors [9].

Significant advances have recently been made in the characterization of the molecular landscape at the basis of TET underlined by The Cancer Genome Atlas (TGCA) [10] data publication and the identification of the non-coding RNA contribution to TET-associated phenotype [11–13].

N6-methyladenosine (m^6^A), one of the most abundant internal modification of mRNA and noncoding RNA (ncRNA), is an important modification involved in RNA splicing [14], translation [15, 16], stability [17] and export [18], leading to modulation of several physiologic and pathologic processes [19–22].

m^6^A modification is catalyzed in the nucleus by a multicomponent complex formed by two methyltransferases METTL3 (MT-A70) and METTL14 (or KIAA1627) [23]. The first one is the catalytic subunit of the complex, while METTL14 has a degenerate active site and plays non-catalytic roles in maintaining complex integrity and substrate RNA binding [24, 25]. Other factors are necessary in vivo to catalyze m^6^A modification, like WTAP, required for complex localization into nuclear speckles [26], VIRMA (KIAA1429) and RBM15 for recruitment of the methyltransferase complex on the target site of the pre-mRNA, ZC3H13 required for RBM15 and WTAP association [27].

This RNA modification is dynamic and reversible; therefore, it can be erased by FTO and ALKBH5 demethylases [23], both belonging to the AlkB deoxygenase family and localized in the nuclear speckles [28, 29]. The first readers to be identified are the members belonging to YT521-B homology (YTH) domain family (YTHDF1, YTHDF2, YTHDF3, YTHDC1 and YTHDC2), which contain an aromatic cage for specifically accommodating the m^6^A [30]. Furthermore, m^6^A can induce a structural modification in transcripts, termed 'the m^6^A-switch’, favoring or inhibiting the interaction with hnRBP [31, 32].

The recent rediscovery of m^6^A is due to its crucial role in the growth and progression of many types of cancer, where different components have an alternated expression, in particular METTL3. Some roles described for the physiological functions of the cell are exploited by cancer cells to their advantage to allow the self-renewal of the cancer stem cells, to increase translation of oncogenes such as EGFR, MYC, TAZ, MAPKAPK2 and to use shelter systems to allow cell survival [33–37]. Moreover, an interplay between m^6^A modification and non-coding RNAs, in particular microRNAs and lncRNAs has recently emerged [38]. MALAT1, for example, a lncRNA frequently upregulated in cancer, contains several m^6^A modifications that impact on its functional activity [39, 40].

In this work, we assessed that METTL3 is upregulated in thymic tumors and we investigated the biological significance of the increased levels of METTL3 in thymic carcinoma cells. Moreover, we evaluated whether METTL3 and its downstream target c-MYC may represent powerful targets to improve the treatment of thymic tumors.

## Results

### METTL3 is upregulated in TETs

**Fig. 1:**
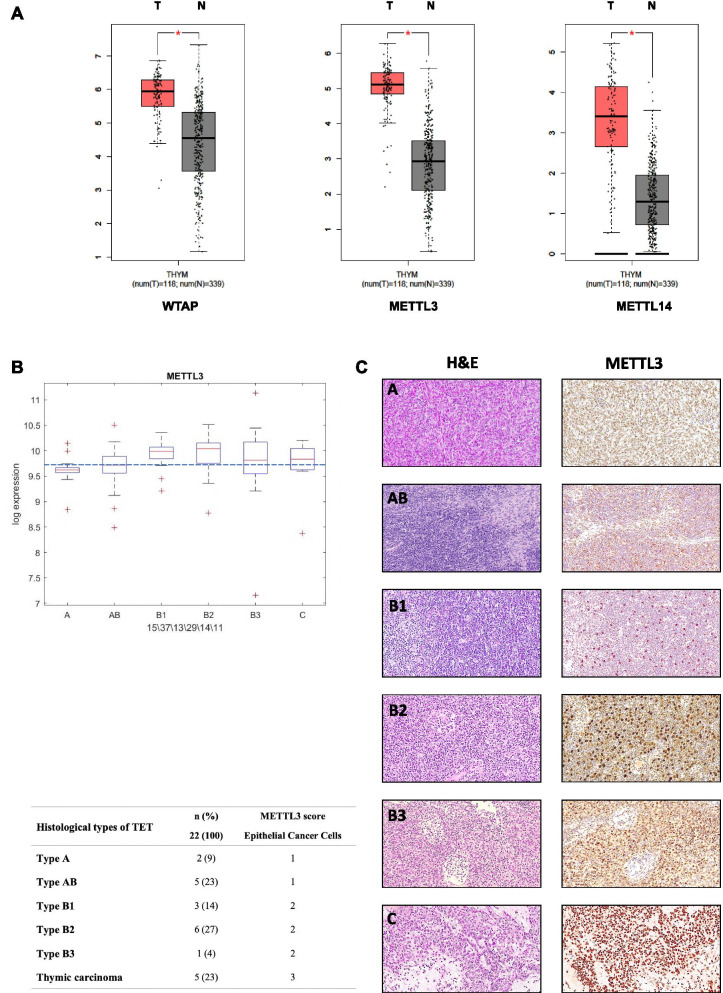
METTL3 is upregulated in thymic carcinoma. **A** Differential expression of WTAP, METTL3 and METTL14 mRNA between normal (N) and thymic carcinoma (T) samples (http://gepia.cancer-pku.cn). **B** METTL3 expression in different types of Thymoma and Thymic carcinoma (TCGA dataset). **C** Immunohistochemistry of METTL3 in different types of Thymoma and Thymic carcinoma. For each representative tissue, hematoxylin and eosin (HE) staining (left) and the corresponding immunohistochemistry (IHC) for METTL3 (right) are shown. The associated table summarizes results of METTL3 IHC in epithelial cancer cells from 22 cases of TET. METTL3 immunostaining intensity was determined as reported in Methods

To explore the possible contribution of components of the protein machinery enabling N6-methyladenosine (m^6^A) methylation of RNAs to thymic cancer, we first took advantage of the thymoma GEPIA dataset to evaluate whether any modulation of these components was present between cancer and normal tissues. As shown in Fig. 1A, we observed that WTAP, METTL3 and METTL14 were significantly upregulated in cancer vs. normal tissues, with METTL3 showing the most significant deregulation. Further analysis of the TCGA dataset showed that median expression of METTL3 mRNA was higher in B1, B2, B3 and C compared to A and AB histotypes (Fig. 1B). To complement the analyses at mRNA level, we analyzed METTL3 protein expression by immunohistochemistry on thymic tumor tissues (*N* = 22). Interestingly, analysis of METTL3 protein staining intensity in epithelial cancer cells revealed an increase of intensity from types A/AB (score of intensity = 1) to types B1-B3 (score of intensity = 2), and reached a maximum of intensity (score = 3) in thymic carcinoma cases (C) (Fig. 1C).

### METTL3 impacts on cell proliferation and translation rate

**Fig. 2:**
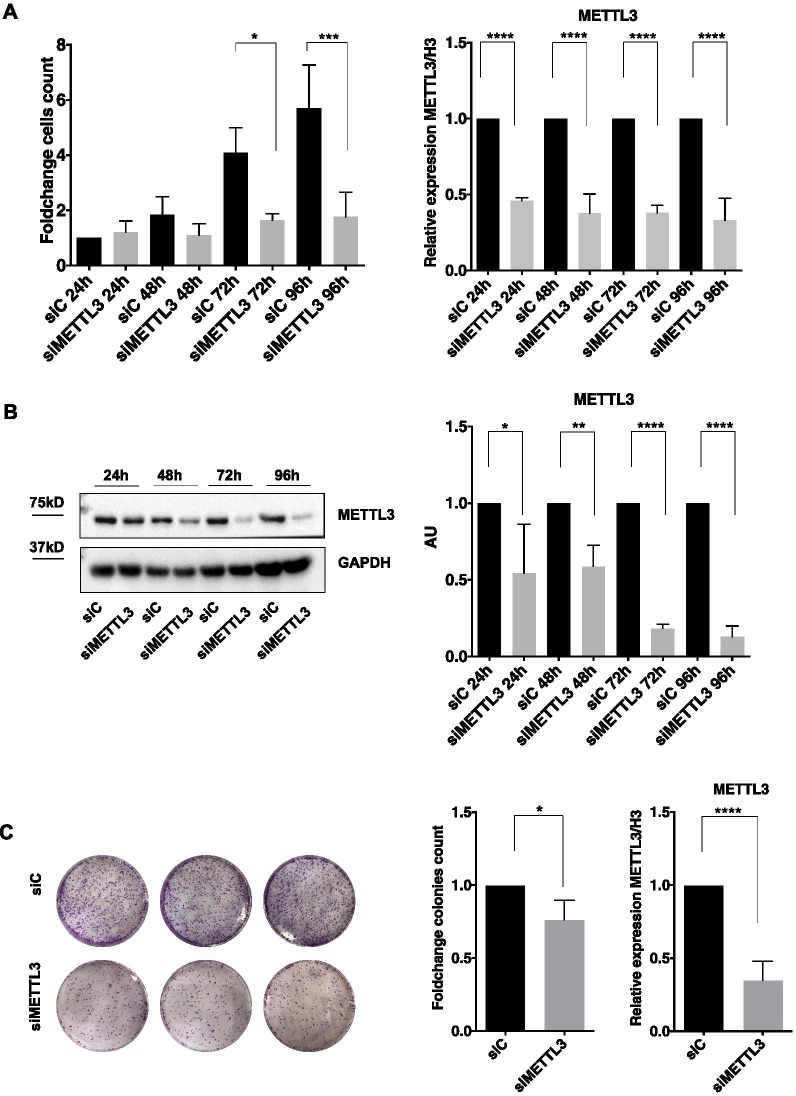
METTL3 silencing reduces cell proliferation and colony formation. **A** Growth curve of TC1889 cells transfected with siC and siMETTL3 and counted with Trypan blue for 24, 48, 72, 96 h (*n* = 3); analysis of METTL3 mRNA expression by RT-qPCR is shown on the right panel at the same time points (*n* = 3). **B** Representative western blot of METTL3 and its relative quantification (*n* = 3). **C** Colony assay of TC1889 cells silenced for METTL3 (72 h), visualized by crystal violet staining after 2 weeks (left) and counted (middle) using ImageJ software (*n* = 4). Efficiency of METTL3 depletion was assessed by RT-qPCR at 72 h from siRNA transfection (right). *p ≤ 0.05; ***p ≤ 0.0005; ****p ≤ 0.00005; P-values have been calculated by two-tailed T-test

To investigate the biological output of the high expression level of METTL3 in TET, we depleted METTL3 by siRNA transfection in the thymic carcinoma cell line TC1889, to evaluate the effects on proliferation and colony forming ability. As shown in Fig. 2A-B, Additional file 1, 2: S1A and S2A, a marked decrease of proliferative potential in cells depleted of METTL3 compared to control cells was observed. Accordingly, TC1889 cells depleted of METTL3 expression for 72 h also showed a significant decrease of colony forming ability (Fig. 2C). We didn't observe any impact of METTL3 depletion on migration capacity (Additional file 1: Fig. S1B), differently from what previously reported in other cell types [41–43].

**Fig. 3:**
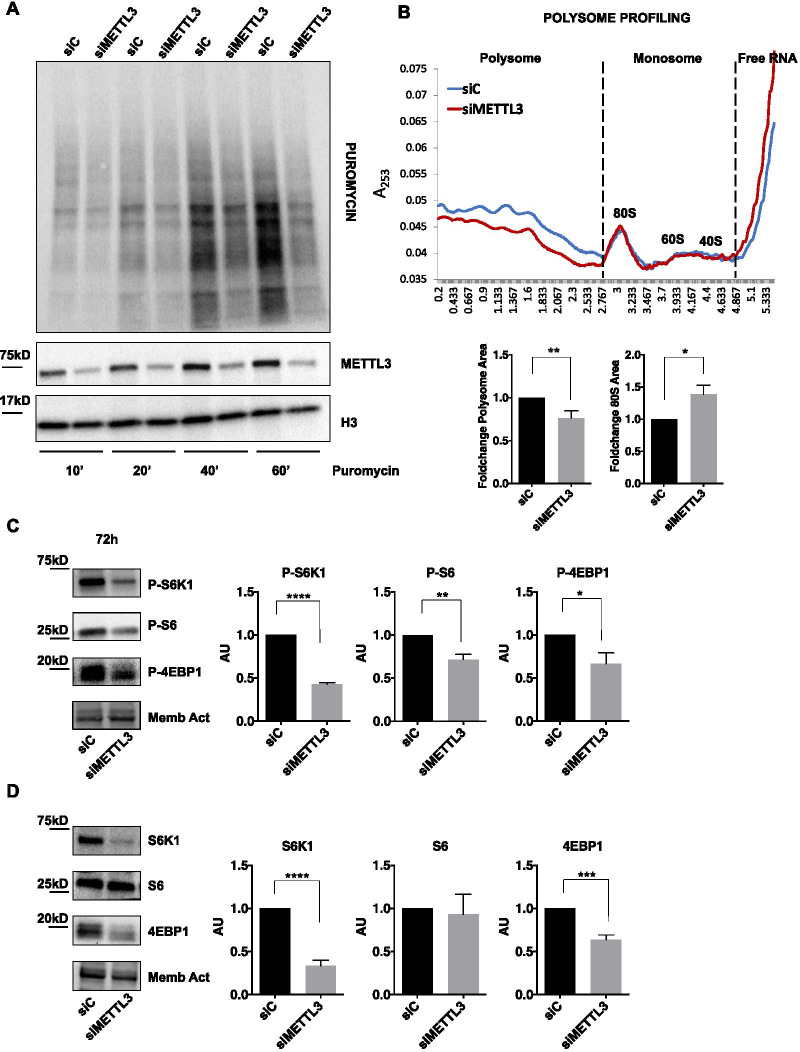
METTL3 enhances translation rate in thymic carcinoma cells. **A** Puromycin incorporation for the indicated time points, detected by western blot analysis in control (siC) and METTL3-silenced (siMETTL3) TC1889 cells after 72 h. See also Additional file 3: Fig. S3 for quantifications (*n* = 4). **B** Representative ribosomal fractionation profiles of control (siC, blue) and 48 h METTL3-silenced (siMETTL3, red) TC1889 cells. On the right, calculation of area under the polysome and 80S curve after 48 h of METTL3 depletion, using ImageJ software (*n* = 3). **C**, **D** Western blot analysis showing the phosphorylated (**C**) and basal (**D**) levels of the indicated translation-related proteins in control (siC) and METTL3-silenced (siMETTL3) TC1889 cells after 72 h. Quantifications of three independent experiments are shown in the right panels. *p ≤ 0.05; **p ≤ 0.005; ***p ≤ 0.0005; ****p ≤ 0.00005; *p* values have been calculated by two-tailed T-test

As METTL3-dependent m^6^A modification may influence the translation rate of targeted mRNAs [16, 44], we wondered whether the observed decreased proliferation rate of METTL3-depleted cells could be attributable to reduced translation potential of cells. To address this, we evaluated the effect of modulation of METTL3 on global translation efficiency in TC1889 cells. Specifically, 72 h METTL3-depleted (siMETTL3) and control (siC) cells were challenged with puromycin, a tyrosyl-tRNA mimic that blocks translation by labeling and releasing elongating polypeptide chains from translating ribosomes, for 10, 20, 40 or 60 min. We measured the levels of newly synthesized polypeptides using an anti-puromycin antibody. We observed a strong decrease of puromycin incorporation upon METTL3 silencing compared to control cells, indicating a global decrease of protein synthesis (Fig. 3A and Additional file 3: Fig. S3A). According to this observation, polyribosome (polysome) fractionation analysis evidenced a drop of polysome fraction in samples depleted of METTL3 for 48 h and 72 h and an increase of 80S ribosome fraction compared to control samples (Fig. 3B and Additional file 3: Fig. S3B).

Analysis of the protein levels of crucial regulators of protein synthesis, such as p70 ribosomal protein S6 kinase 1 (S6K1), its substrate ribosomal protein S6 (S6), and eukaryotic translation initiation factor 4E-binding protein 1 (4EBP1), revealed that METTL3 depletion causes a marked and significant decrease of the phosphorylated forms of these upstream regulators, compared to control cells (Fig. 3C), indicating a role for METTL3 in the induction of translation in TET cells. Interestingly, METTL3 depletion also caused a decrease of the basal level of expression of translation regulators S6K1 and 4EBP1, while no changes in S6 levels were detected (Fig. 3D).

### METTL3 regulates c-MYC mRNA and protein levels

It has been extensively reported that c-MYC, a well characterized oncogene involved in the induction of cell proliferation and growth, is targeted by METTL3 in numerous malignancies, including acute myeloid leukemia (AML) [33, 45, 46]. We then explored whether the METTL3-dependent increase of cell proliferation and translation rate in TET could be attributed to the induction of c-MYC.

**Fig. 4:**
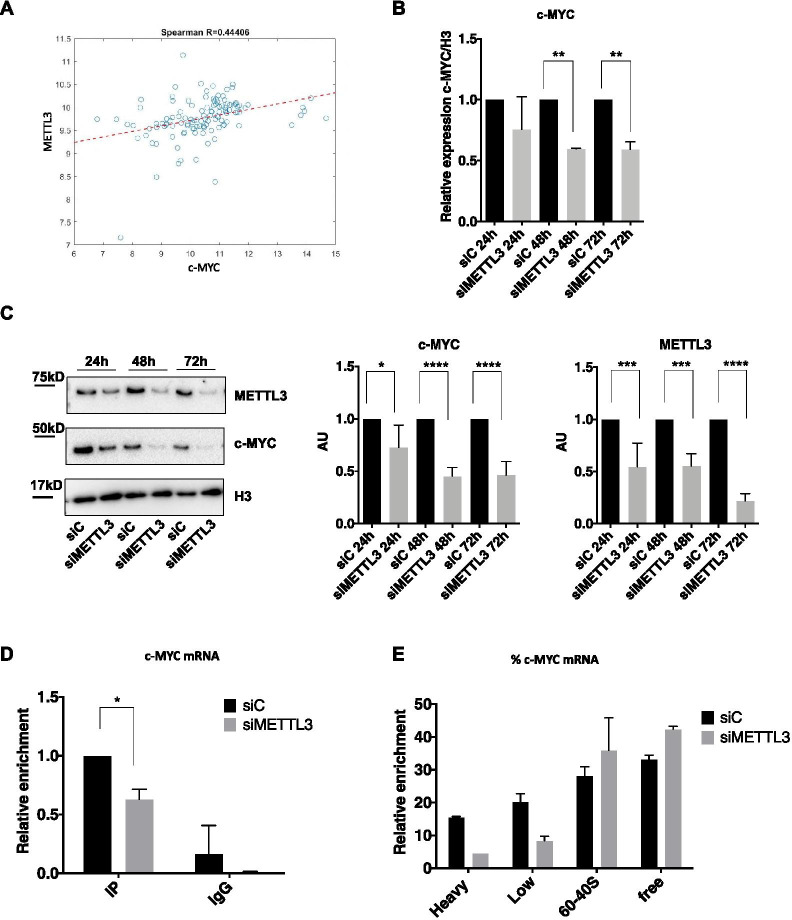
METTL3 controls c-MYC expression. **A** Spearman’s correlation plot showing the expression of METTL3 and c-MYC mRNAs in Thymoma and Thymic carcinoma samples from the TCGA dataset. **B** RT-qPCR analysis of c-MYC at 24 h, 48 h, 72 h of METTL3 silencing in TC1889 cells (*n* = 3). **C** Representative western blot analysis of c-MYC and METTL3 proteins at 24 h, 48 h and 72 h after silencing of METTL3 in TC1889 cells (left). Quantifications of four independent western blots are shown in middle (c-MYC) and right (METTL3) panels. **D** Immunoprecipitation was performed using an antibody recognizing m^6^A modification (IP) or IgG as negative control, followed by RT-qPCR analysis of c-MYC on recovered RNA samples, in control (siC) and METTL3-silenced (siMETTL3) TC1889 cells (*n* = 2). **E** Distribution of c-MYC mRNA, evaluated by RT-qPCR analysis, in ribosomal fractions obtained from control (siC) and METTL3-silenced (siMETTL3) TC1889 cells (48 h). *p ≤ 0.05; **p ≤ 0.005; *p*-values have been calculated by two-tailed T-test

Bioinformatic analysis of TGCA data showed a positive correlation between METTL3 and c-MYC mRNA levels in Thymoma and Thymic carcinoma samples (Fig. 4A). mRNA (Fig. 4B) and protein (Fig. 4C and Additional file 2: Fig. S2B) expression analysis of c-MYC in TC1889 cells showed a significant reduction of c-MYC expression in METTL3-depleted cells compared to control cells. m^6^A immunoprecipitation confirmed that c-MYC is a target of METTL3 methyltransferase activity, as silencing of METTL3 led to a 40% reduction of c-MYC mRNA methylation (Fig. 4D). The marked decrease of c-MYC protein level in METTL3-depleted cells led us to investigate the translation rate of c-MYC to uncover a possible role of METTL3 in c-MYC translation. After 48 h of METTL3 depletion, analysis of c-MYC transcript in samples from polysome fractionation revealed a shift of distribution from the heavier to the lighter polysome fractions occurring in METTL3-depleted cells compared to control (Fig. 4E and Additional file 3: Fig. S3C). Altogether, these results indicate that METTL3 controls c-MYC transcript and protein level also in TET.

### METTL3-dependent m^6^A modification of MALAT1 impinges on MYC protein synthesis

**Fig. 5:**
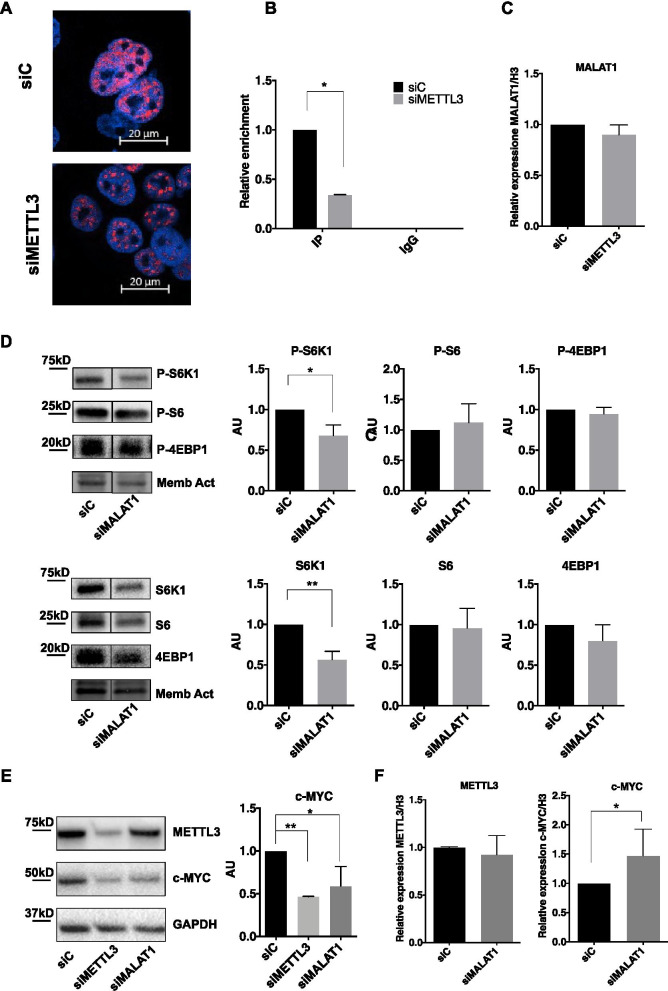
MALAT1 is delocalized following METTL3 depletion and regulates c-MYC protein expression. **A** Analysis of MALAT1 subcellular localization by RNA FISH in control (siC) and METTL3-silenced (siMETTL3) TC1889 cells (72 h). **B** Immunoprecipitation was performed using an antibody recognizing m^6^A modification (IP) or IgG as negative control, followed by RT-qPCR analysis of MALAT1 on recovered RNA samples, in control (siC) and METTL3-silenced (siMETTL3) TC1889 cells (*n* = 2). **C** Analysis by RT-qPCR of MALAT1 expression in control (siC) and METTL3-silenced (siMETTL3, 72 h) TC1889 cells (*n* = 3). **D** Western blot analysis showing the phosphorylated (upper panel) and basal (lower panel) levels of the indicated translation-related proteins in control (siC) and MALAT1-silenced (siMALAT1) TC1889 cells (72 h). Quantifications of three independent experiments are shown in the right panels. **E** Western blot analysis showing METTL3 and c-MYC protein level in TC1889 cells depleted of METTL3 or MALAT1 by siRNA transfection. Quantification of three independent experiments is shown on the right. **F** RT-qPCR analysis of METTL3 and c-MYC expression in control (siC) and MALAT1-depleted (siMALAT1) TC1889 cells (*n* = 3). *p ≤ 0.05; **p ≤ 0.005; *p*-values have been calculated by two-tailed T-test

We next explored potential mediators responsible for METTL3-dependent increased translation of c-MYC. Since it has been reported that long non-coding RNA MALAT1 may be subjected to m^6^A modification and may control translation in various malignancies, we investigated whether its activity was regulated by METTL3. To this end, we first evaluated by RNA FISH the subcellular localization of MALAT1 in control and METTL3-depleted TC1889 cells. It has been indeed reported that MALAT1 may be localized in nuclear speckles and/or it may be diffused in the nucleoplasm [47, 48], and that different localization is associated with specific functions. As shown in Fig. 5A and Additional file 2: Fig. S2C, while MALAT1 showed a quite diffused staining in controls TC1889 cells (siC), it acquired a clear-cut localization in nuclear speckles in METTL3-depleted cells (72 h). This correlated with significant decrease in m^6^A modification of MALAT1 RNA (Fig. 5B) without any significant decrease in overall MALAT1 expression (Fig. 5C). On the basis of METTL3-dependent modification of MALAT1 m^6^A modification and subnuclear localization, we explored whether depletion of MALAT1 impinges on the METTL3-associated effects on translation rate and c-MYC expression. As shown in Fig. 5D, MALAT1 depletion for 72 h by siRNA transfection (Additional file 4: Fig. S4A) led to a significant reduction of both S6K1 protein level and its phosphorylated form, while no changes in S6 and 4EBP1 were present. Interestingly, depletion of MALAT1 also caused a marked reduction of c-MYC protein (Fig. 5E). We have also evaluated, in the same experimental conditions as above, the expression level of VEGFA isoforms, which we have previously reported to be affected by MALAT1 subnuclear localization in breast cancer cells [48]. Expression of both VEGF165 and VEGF121 is decreased following MALAT1 or METTL3 depletion as shown in Additional file 4: Fig. S4B. Next we evaluated the impact of single or combined METTL3 and MALAT1 silencing on c-MYC expression. To address this, we considered the 24 h time point of METTL3 silencing, when c-MYC protein is not yet strongly decreased (as already shown in Fig. 4C). Combined METTL3 and MALAT1 silencing led to a more marked reduction of c-MYC protein expression compared to single silencing (Additional file 4: Fig. S4C). Interestingly, silencing of METTL3 decreases c-MYC mRNA and protein, while MALAT1 silencing impinges only on c-MYC protein level (Fig. 5F).

### Silencing of METTL3 combined with cisplatin or c-MYC inhibitor induces cell death in TET cells

Surgery, preceded and/or followed by chemotherapy and radiotherapy, is currently the gold standard for the treatment of thymic epithelial tumors.

**Fig. 6:**
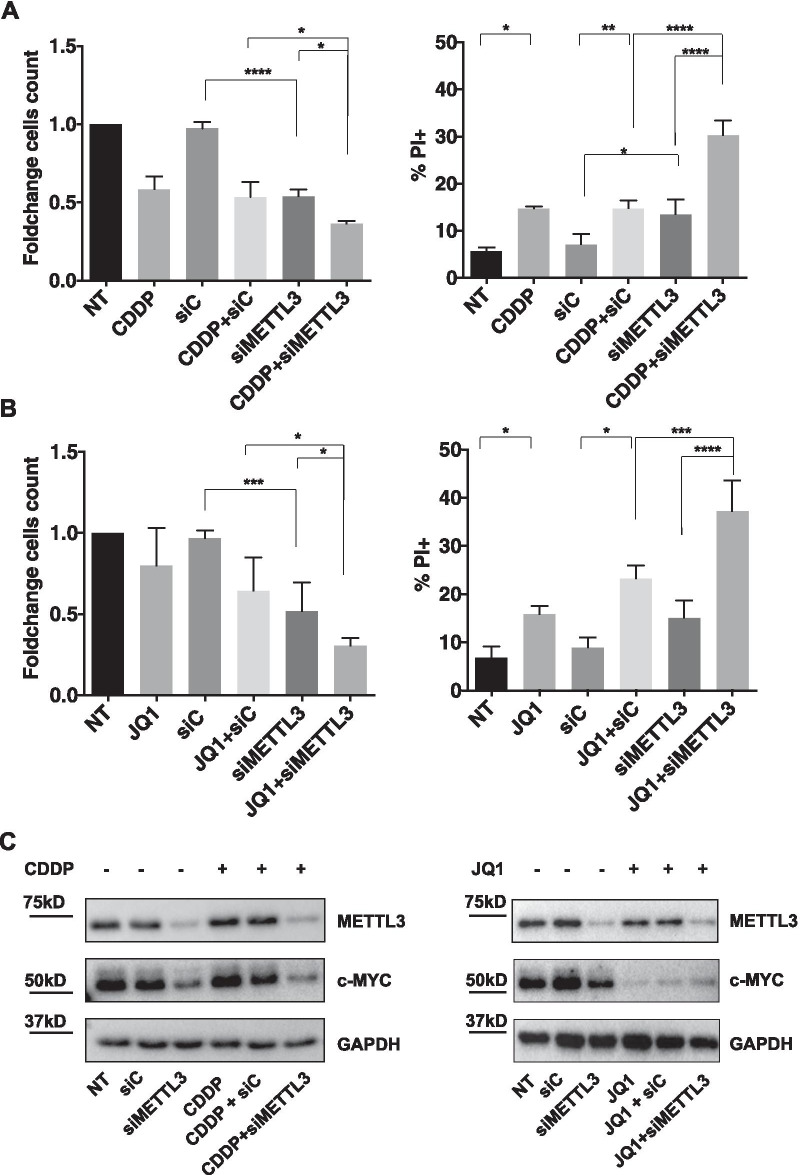
METTL3 depletion cooperates with cisplatin or JQ1 inhibitor in the induction of cell death. **A**–**B** Cell counting analysis by trypan blue exclusion (left) and cytofluorimetric analysis (right), after 48 h of treatment with CDDP (**A**) or JQ1 (**B**), in control (siC) and METTL3-silenced (siMETTL3) TC1889 cells, *n* = 3. **C** Representative Western blot analysis of METTL3 and c-MYC proteins in cells treated as in (**B**, **B**). See also Additional file 5: Fig. S5 for quantifications. *p ≤ 0.05; **p ≤ 0.005; ***p ≤ 0.0005; ****p ≤ 0.00005; *p*-values have been calculated by One-Way Anova

To investigate whether METTL3 upregulation in TET impinges on the response to chemotherapy, we evaluated whether TC1889 cells silenced for METTL3 showed different sensibility to cisplatin, compared to control cells. Specifically, we monitored cell death and proliferation in cells transfected with control or METTL3 siRNAs and subsequently treated with cisplatin (CDDP, 7.5 μg/mL). We observed that CDDP treatment or METTL3 depletion significantly induced cell death and that the combination of CDDP and METTL3 silencing determined an additive effect, compared to individual treatments, leading to reduced proliferation (Fig. 6A, left) and increased cell death (Fig. 6A, right). This last effect was also confirmed by the analysis of the ratio between Cleaved-PARP and PARP (Additional file 5: Fig. S5B). Analysis of c-MYC protein level in the same experimental condition is shown in Fig. 6C (left) and Additional file 5: Fig. S5A.

On the basis of METTL3-dependent c-MYC protein induction, we evaluated the effect of the combination of METTL3 depletion and c-MYC inhibition on cell death and proliferation. c-MYC was inhibited using the small molecule JQ1, a selective bromodomain inhibitor able to block the c-MYC inducer BRD4 [49–52]. 24 h after transfection with siRNAs siC or siMETTL3, we treated cells with JQ1 (3 μM) for additional 48 h. We observed reduced proliferation and increased cell death in the combined compared to individual treatment (Fig. 6B and Additional file 5: Fig. S5B). Analysis of c-MYC protein level in the same experimental condition is shown in Fig. 6C (right) and Additional file 5: Fig. S5A.

This result suggests that a block of c-MYC cooperates with METTL3 depletion, impinging on cell viability and proliferation, and highlighting this axis as a promising target to be exploited for the treatment of TET.

## Discussion

This study focused on the study of a specific enzyme, METTL3, and its role in Thyimic epithelial tumors (TETs). METTL3, the main component of the m^6^A methyltransferase complex, plays a crucial role in many biological processes especially in tumorigenesis, either dependently on or independently from its m^6^A RNA methyltransferase activity[53, 54].

As described in a variety of studies [55, 56], METTL3 is expressed at higher level in tumor tissues compared to normal and we demonstrate here that it is up-regulated also in TETs tissues. As the role of METTL3 in Thymoma and Thymic carcinoma is currently uncharacterized, we first analyzed the effects of METTL3 silencing on the thymic tumor phenotype, using TC1889 cells obtained from a Thymic carcinoma, highlighting the requirement of METTL3 expression for proliferation and high translation rate. At molecular level, METTL3 is responsible for the high expression and phosphorylation of crucial proteins which control translation, such as the mTOR-dependent S6K1, S6 and 4EBP1. Specifically, silencing of METTL3 decreases the basal and phosphorylated forms of S6K while reducing only the phosphorylated form of S6; this suggests that reduction of P-S6 is a consequence of S6K modulation by METTL3. It has been previously proposed that METTL3 activity is fundamental for normal cell survival; an important limitation of this study is represented by the lack of normal thymic cells analysis upon METTL3 depletion. Further studies focused on normal thymic epithelial cells will clarify whether or not the identified networks are specific to TET cells.

One of the most well-known oncogenes and key regulator of cell proliferation is c-MYC. Several reports have demonstrated that abnormal expression of c-MYC may be related to increased m^6^A modification of its mRNA in several tumors as for example in thyroid cancer [57], or in oral squamous cell carcinoma (OSCC) [46]. Consistent with these evidences, we validated c-MYC as target of METTL3-dependent methylation also in Thymic carcinoma and showed the requirement of METTL3 for an efficient c-MYC expression in TC1889 cells, suggesting that METTL3 might enhance cell proliferation by promoting c-MYC translation. Accordingly, a positive correlation between METTL3 and c-MYC expression was observed by analyzing the TCGA TET dataset. Increased c-MYC expression had been already reported in TET. Specifically, the TCGA study revealed an upregulation of the oncogenes MYC/MAX in AB-, B-, and C-like associated molecular signatures compared to A-like subtype, in line with their known increased clinical aggressiveness in TETs [10].

Recently, different studies have reported that m^6^A modifications can also regulate the biogenesis and function of noncoding RNAs, such as miRNAs, lncRNAs, and circRNAs [38]. In this study we found that MALAT1 is an additional target of METTL3-dependent methylation, and that this modification is able to influence the subnuclear localization of this lncRNA, leading to a diffused nucleoplasmic staining; on the contrary, MALAT1 shows a clear-cut localization in nuclear speckles in METTL3-depleted cells. Interestingly, similarly to METTL3-depleted cells, MALAT1-depleted TC1889 showed decreased S6K1 protein level (including its phosphorylated form). However, siMALAT1 causes a less marked decrease of S6K and P-S6K, compared to siMETTL3, suggesting that this could be not sufficient to drive the S6 dephosphorylation observed in siMETTL3. It's also worth underlining that MALAT1 depletion by siRNA transfection isn't exactly equivalent to METTL3 depletion, which causes a relocalization of MALAT1 in nuclear speckles. This could also explain the incomplete overlap of results in the two interference conditions.

Similarly, to METTL3-depleted cells, MALAT1-depleted TC1889 also showed decreased c-MYC protein, suggesting that METTL3 could regulate c-MYC translation also through the delocalization of MALAT1. However, silencing of MALAT1 didn't impact on the m^6^A modification of c-MYC mRNA (data not shown), suggesting that it is not required for METTL3 enzymatic activity.

Previously, we have shown that an alternative oncogenic stimulus (e.g. the presence of mutated p53 protein and overexpressed ID4) causes a similar delocalization of MALAT1, in breast cancer cells, with consequent altered splicing [48]. The possibility that m^6^A modification could mediate also the effects previously observed in breast cancer cells is certainly an intriguing possibility that merits to be verified in future studies. Interestingly, it has been also recently reported that m^6^A modification of MALAT1 is critical for metastatic ability of cancer cells, reshaping the nuclear speckles [58].

Although the gold standard treatment for thymic epithelial tumors remains surgery, the use of several innovative agents for TET treatment is under investigation [59]. For this reason, we analyzed and established a sensitization effect of thymic carcinoma cells to a chemotherapeutic agent, like cisplatin, upon METTL3 silencing. As c-MYC overexpression has been extensively reported as a feature conferring chemoresistance to cancer cells, we can hypothesize that the reduced c-MYC protein levels observed after METTL3 silencing could represent the determinant allowing sensitization to cisplatin treatment. Of note, METTL3 depletion similarly sensitizes cells to the treatment with JQ1, a BRD4 inhibitor impairing c-MYC expression.

## Conclusions

In conclusion, our study identified the oncogenic role of METTL3 also in Thymic Ephitelial Tumors, which improves cells proliferation trough the control of c-MYC expression. Moreover, our results highlight METTL3 as a powerful target whose depletion improves the response to different anticancer treatments and open to the possibility of exploiting this protein to improve the treatment of TETs.

## Methods

### Cell culture, transfection and treatment

Human Thymic Carcinoma cell line TC1889, provided by Prof. Ralf J Rieker [60], was cultured with RPMI 1640 (Gibco® Thermo Fisher Scientific, Waltham, MA USA) enriched with glucose (4.5 g/L), Hepes (25 mM), pen/strep (50 U/mL) and with 10% heat-inactivated South-American Fetal Bovine Serum (FBS) (Gibco® Thermo Fisher Scientific, Waltham, MA, USA), at 37 °C and 5% CO2.

TC1889 cells were transfected with siRNAs for METTL3 (#SI04140038, #SI04241265, #SI04317096, #SI04340749, Qiagen Chatsworth, CA), MALAT1 [47] and control (#1022076, Qiagen Chatsworth, CA) at final concentration of 5 nM, using Lipofectamine RNAiMAX (Gibco® Thermo Fisher Scientific, Waltham, MA, USA) according to the manufacturer’s instructions. For METTL3 silencing a pool of 4 siRNA sequences was used. siRNAs sequences are: siC, AATTCTCCGAACGTGTCACGT; siMETTL3_5, CCGCGTGAGAATTGGCTATAT; siMETTL3_6, CAGGAGATCCTAGAGCTATTA; siMETTL3_7, CTGCAAG TATGTTCACTATGA; siMETTL3_8, AGGAGCCAGCCAAGAAATCAA; siMALAT1_1, GATCCATAATCGGTT TCAA. Double depletion of METTL3 and/or MALAT1 and/or siC was performed transfecting TC1889 cells with siC and siMALAT1 for 48 h and then with siC and siMETTL3 for additional 24 h.

After 24 h of METTL3 silencing, TC1889 cells were treated with Cisplatin (CDDP, 7.5 μg/mL) (#S1166 Selleck Chemicals, Italy), for additional 48 h. The same experiment was performed with ( +)-JQ1 (3 μM, #SML1524, Sigma-Aldrich, USA) treatment.

### Immunohistochemistry

METTL3 protein expression was analyzed by immunohistochemistry (IHC) in a set of 22 Formalin fixed, paraffin embedded (FFPE) thymic epithelial tumors of different histotypes derived from the Pathology Department of IRCCS Regina Elena National Cancer Institute of Rome, Italy. The thymomas FFPE samples included: 2 type A, 5 type AB, 3 type B1, 6 type B2, and 1 type B3. Moreover, 5 FFPE carcinoma samples were analyzed. Thymomas and thymic carcinoma samples from “Policlinico Umberto I” Hospital of Rome, Italy were used to set up the experimental condition. This study was approved by the Institutional Review Board of “Policlinico Umberto I” Hospital (Rif 3262/26.06.2014 Prot. N° 815/14) and the Regina Elena National Cancer Institute (Rif 383/28.5.2013 Prot. N°5/13 and Rif 447/20.06.2013 Prot. N°7/13). In brief, 5 μm-thick sections from each sample were Haematoxylin and Eosin stained, and consecutive sections were stained with the anti-METTL3 antibody (#ab195352, Abcam). The METTL3 immunostaining intensity was determined according to Liang et al., 2020 [61], and the staining intensity was scored as follows: no staining = 0; weak staining = 1; moderate staining = 2; strong staining = 3.

### Clonogenic assays

Cells were detached 72 h after METTL3 silencing and seeded into 6-well dishes at a concentration of 1 × 10^3^ cells/well. Medium was changed every four days. 10–14 days after plating, cells were washed with PBS for three times and were stained using crystal violet dye. Cells were counted using ImageJ software.

### RNA extraction and real-time qRT-PCR analysis

Total RNA was extracted using the TRIzol RNA Isolation System (Invitrogen) according to manufacturer instructions. Reverse transcription to cDNA was performed with the High-Capacity RNA-to-cDNA Kit (Applied Biosystems) and cDNA was amplified using the SYBR™ Green PCR Master Mix (Thermo Fisher Scientific, Waltham, MA USA) on an ABI PRISM 7500 Sequence Detection System (Applied Biosystems). Relative expression levels of targets were determined with the comparative 2^−ΔΔCt^ method, using H3 as control. All reactions were performed in duplicate. The following oligo sequences were used: METTL3 For, AAGCAGCTGGACTCTCTGCG; METTL3 Rev, GCACTGGGCTGTCACTACGG; c-MYC For,AGCTGCTTAGACGCTGGATT; c-MYC Rev, AAGTTCTCCTCCTCGTCGC; MALAT1 For, GAGGTCTTTGGTGGGTTGAA; MALAT1 Rev, CCCACCCAGCATTACAGTTC; H3 For, GTGAAGAAACCTCATCGTTACAGGCCTGGT; H3 Rev, CTGCAAAGCACCAATAGCTGCACTCTGGAA.

### Lysis and immunoblotting analysis

Cells were lysed in 2% SDS buffer (25 mM Tris–Hcl pH 7.5, 100 mM Nacl, 3 mM EDTA, 7% Glycerol) and fresh protease inhibitors (PMSF 1 mM, NaF 1 mM, NaVO3 1 mM, Na4P207 5 Mm, Apoprotein 2 μg/ml, Leupeptin 5 μg/ml). Then, cells were sonicated for 10 s and centrifuged for 10 min at 12,000 × rpm. Lysates were quantified using Bradford Assay Reagent (#1863028, Thermo Fisher, USA). Protein samples were separated by SDS-PAGE and transferred onto a nitrocellulose membrane. The membrane was incubated overnight with the following primary antibodies: rabbit monoclonal anti-METTL3 (#ab195352, Abcam); rabbit monoclonal anti-H3 (#ab1791, Abcam); rabbit monoclonal anti-c-MYC (#5605, Cell Signaling Technology); mouse monoclonal anti-Puromycin, clone 12D10 (#MABE343, Millipore); rabbit monoclonal anti-p70 S6 (#9202, Cell Signaling Technology); rabbit monoclonal anti-phospho p70 S6 Kinase (thr389; #9234, Cell Signaling Technology); mouse monoclonal anti-S6 (#2317, Cell Signaling Technology); rabbit monoclonal anti-phospho S6 (ser235/236; #2211, Cell Signaling Technology); rabbit monoclonal 4EBP1 (#9452, Cell Signaling Technology); rabbit monoclonal anti-phospho 4EBP1 (#2855, Cell Signaling); rabbit monoclonal anti-PARP (46D11; #9532, Cell Signaling); mouse monoclonal anti-GAPDH 6C5 (#sc32233, Santa Cruz Biotechnology); mouse monoclonal anti-Tubulin B512 (#T5168, Sigma-Aldrich, Milan, Italy). For the detection we used ECL method (Enhanced ChemiLuminescence) (Amersham Biosciences) using ChemiDoc-It Imaging System (UVP, Upland, CA) instrument.

### Polysome profiling and treatment with puromycin

After 48 h of transfection with METTL3 and Control siRNA, 20 × 10^6^ cells were incubated 10 min with cycloheximide (Sigma-Aldrich) and then lysed with 400 μL of lysis buffer (10 mM Tris pH 7.5, 10 mM NaCl, 10 mM MgCl2, 1% Triton X-100) supplemented with 10 mM fresh DTT, 100 μg/ml cycloheximide, 1X PIC (Complete, EDTA free, Roche) and 1X RNase guard (Thermo Scientific). Cells were allowed to swell for 10 min on ice and the lysates were centrifuged for 10 min at 13,000 rpm at 4 °C. The supernatants were collected, loaded on 15–50% sucrose gradient and centrifuged at 38,000 rpm with a SW41 rotor (Beckman) for 1 h 30 min at 4 °C. Fractions were collected with a Bio-logic LP (Biorad). Then, 200 μL of each fraction were pooled together 3 by 3 obtaining four fractions (Heavy Polysomes, Light Polysomes, 40S/60S, Free RNA) and total RNA was extracted using Acid Phenol: CHCl_3_ (#AM9720, Ambion, USA) and RNeasy Mini Kit (#74,104, Qiagen, Hilden, Germany).

TC1889 cells were treated with puromycin (#ant-pr-1, InvivoGen) at 10ug/ml after 72 h of transfection with siC or siMETTL3. Incorporated puromycin protein level was then analyzed 10, 20, 40 and 60 min after treatment by Western Blot analysis.

### m^6^A immunoprecipitation

Cells were seeded and transfected with METTL3 and Control siRNA. After 72 h, total RNA was extracted and fragmented into ~ 100 nt long fragments in Fragmentation Buffer (100 mM Tris–HCl and 100 mM ZnCl_2_) for 5′ at 94 °C. A fraction of fragmented RNA was collected as input control and the rest was incubated with m^6^A-specific antibody (#ab151230, Abcam) or with control rabbit IgG (Millipore) antibody for 2 h at 4 °C on rotator. Immunoprecipitated RNA was eluted and resuspended in RNase-free water. RT-qPCR on immunoprecipitated RNA was performed with primers MYC_FW and MYC_REV.

### FISH

Fluorescence in situ hybridization to detect MALAT1 lncRNA was performed using a mixture of 48 fluorescent (Quasar® 570) Stellaris™ RNA probes (#SMF-2035–1, Biosearch Technologies, Inc.). Cells were fixed with 3.7% formaldehyde in PBS and permeabilized with 70% ethanol overnight. Then, the manufacturer's protocol was followed. MALAT1 distribution was visualized with Confocal microscope.

### Statistical analysis

The statistical analyses were performed using GraphPad Prism (GraphPad Software, San Diego, California, USA). Statistical analysis to determine significance was performed using Student’s *t* tests or One-Way Anova test. Differences were considered statistically significant at the *p* < 0.05 level.

## Supplementary Information

**Additional file 1**. **Supplementary Figure 1.** A) After 4 h incubation with 3-(4,5-dimethylthiazol-2-yl)-2,5 diphenyltetrazolium bromide (MTT), METTL3 depleted samples showed a lower cell viability than control samples, in particular at 72 h and 96 h (left panel) (n = 2, triplicates). METTL3 silencing in MTT samples (left panel). B) TC1889 cells after silencing of METTL3 for 72 h were used for Transwell migration assay (24 h of migration). We didn’t observe a decrease in migratory capacity in METTL3 silenced cells compared to control samples. On the right qRT-PCR analysis of METTL3 is shown. (n = 2).

**Additional file 2**. **Supplementary Figure 2.** A) TC1889 cells were transfected with siC, siMETTL3-6, siMETTL3-7 and siMETTL3 (a pool of four siRNA) and counted with Trypan blue at 96 h (n = 3). B) Representative WB of c-MYC and METTL3 after 24, 48, 72 and 96 h of METTL3-6, METTL3-7 and METTL3 depletion. Quantifications of three independent experiments are shown in the bottom panel. C) Analysis of MALAT1 subcellular localization by RNA FISH in control (siC) and METTL3-silenced (siMETTL3-6, siMETTL3-7 and siMETTL3) samples at 72 h.

**Additional file 3**. **Supplementary Figure 3.** A) Puromycin and METTL3 quantification in the samples treated with Puromycin. The samples were normalized on membrane activation lanes (n = 4). B) Representative ribosomal fractionation profile after 72 h of control (siC, blue) and METTL3 silencing (siMETTL3, red) in TC1889 cells. On the right, calculation of area under the polysome and 80S curve in the siC and siMETTL3 samples at 72 h using ImageJ software (n = 2). C) Representative western blot of polysome after 48 h of METTL3 silencing. On the right c-MYC and METTL3 quantification, normalized on GAPDH (n = 3).

**Additional file 4**. **Supplementary Figure 4.** A) Malat1 expression after 72 h of silencing (n = 3). B) VEGF121 and VEGF165 expression was analyzed by qRT-PCR after 72 h of METTL3 and MALAT1 depletion in TET cells (n = 3). C) Representative Western blot of c-MYC after 24 h of METTL3 depletion (siC+siMETTL3 sample), 72 h of MALAT1 depletion (siC+siMALAT1 sample) and 24 h of METTL3 and 72 h of MALAT1 depletion (siMETTL3+siMALAT1 sample). On the top is represented c-MYC and METTL3 quantification, normalized on Membrane Activation (c-MYC n = 4, METTL3 n = 3), while on the bottom, c-MYC and MALAT1 expression was analyzed by qRT-PCR (n = 3).

**Additional file 5**. **Supplementary Figure 5.** A) METTL3 and c-MYC protein levels in TC cells treated with CDDP (top) and JQ1 (bottom) for 48 h. The treatment was performed 24 h after METTL3 silencing (n = 3). B) Representative Western Blot of Cleaved-PARP and PARP and on the right the ratio between Cleaved-PARP and PARP (n = 3).

## Abbreviations

m^6^A: N6-methyladenosine
TETs: Thymic ephitelial tumors
THY: Thymoma
TC: Thymic carcinoma
PAC: Cisplatin, doxorubicin and cyclophosphamide
TGCA: The Cancer Genome Atlas
GEPIA: Gene Expression Profiling Interactive analysis
lncRNA: Long-non-conding RNA
CDDP: Cisplatino
OSCC: Oral squamous cell carcinoma
