# Supplementary material for: METTL3-dependent MALAT1 delocalization drives c-Myc induction in thymic epithelial tumors
Source: Clin Epigenetics. 2021 Sep 16;13:173. doi: 10.1186/s13148-021-01159-6 (PMC8447796; doi:10.1186/s13148-021-01159-6)
Supplement: Supplementary file 1 — Additional file 1. Supplementary Figure 1. A) After 4 h incubation with 3-(4,5-dimethylthiazol-2-yl)-2,5 diphenyltetrazolium bromide (MTT), METTL3 depleted samples showed a lower cell viability than control samples, in particular at 72 h and 96 h (left panel) (n = 2, triplicates). METTL3 silencing in MTT samples (left panel). B) TC1889 cells after silencing of METTL3 for 72 h were used for Transwell migration assay (24 h of migration). We didn’t observe a decrease in migratory capacity in METTL3 silenced cells compared to control samples. On the right qRT-PCR analysis of METTL3 is shown. (n = 2). [file 13148_2021_1159_MOESM1_ESM.pdf]

Figure S1

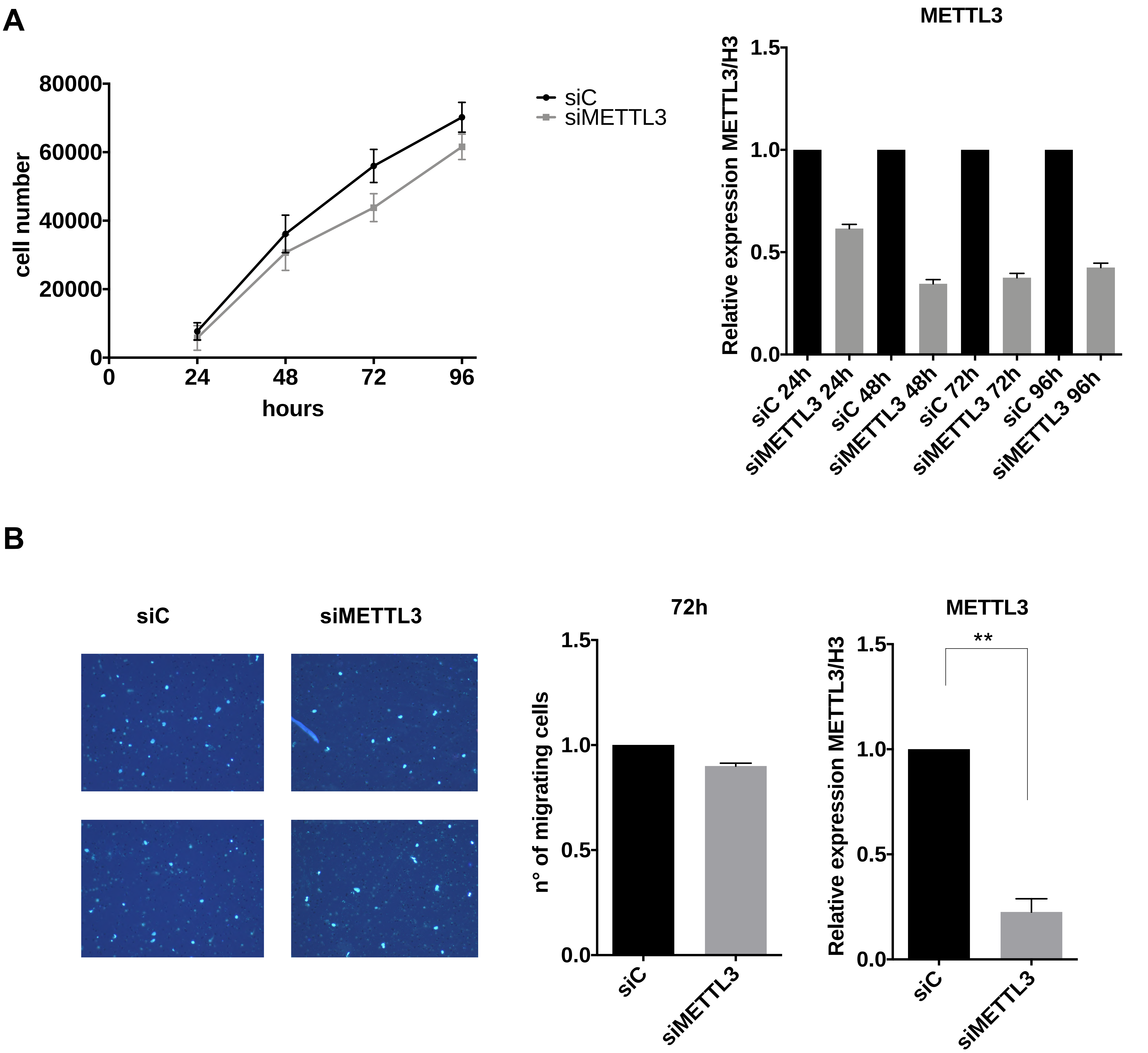

Suppl. Fig. 1. A After 4 hours incubation with 3-(4,5-dimethylthiazol-2-yl)-2,5 diphenyltetrazolium bromide (MTT), METTL3 depleted samples showed a lower cell viability than control samples, in particular at 72h and 96h (left panel) (n=2, triplicates). METTL3 silencing in MTT samples (left panel) . **B** TC1889 cells after silencing of METTL3 for 72 h were used for Transwell migration assay (24 h of migration). We didn't observe a decrease in migratory capacity in METTL3 silenced cells compared to control samples. On the right qRT-PCR analysis of METTL3 is shown. (n=2)
