# Supplementary material for: METTL3-dependent MALAT1 delocalization drives c-Myc induction in thymic epithelial tumors
Source: Clin Epigenetics. 2021 Sep 16;13:173. doi: 10.1186/s13148-021-01159-6 (PMC8447796; doi:10.1186/s13148-021-01159-6)
Supplement: Supplementary file 2 — Additional file 2. Supplementary Figure 2. A) TC1889 cells were transfected with siC, siMETTL3-6, siMETTL3-7 and siMETTL3 (a pool of four siRNA) and counted with Trypan blue at 96 h (n = 3). B) Representative WB of c-MYC and METTL3 after 24, 48, 72 and 96 h of METTL3-6, METTL3-7 and METTL3 depletion. Quantifications of three independent experiments are shown in the bottom panel. C) Analysis of MALAT1 subcellular localization by RNA FISH in control (siC) and METTL3-silenced (siMETTL3-6, siMETTL3-7 and siMETTL3) samples at 72 h. [file 13148_2021_1159_MOESM2_ESM.pdf]

Figure S2

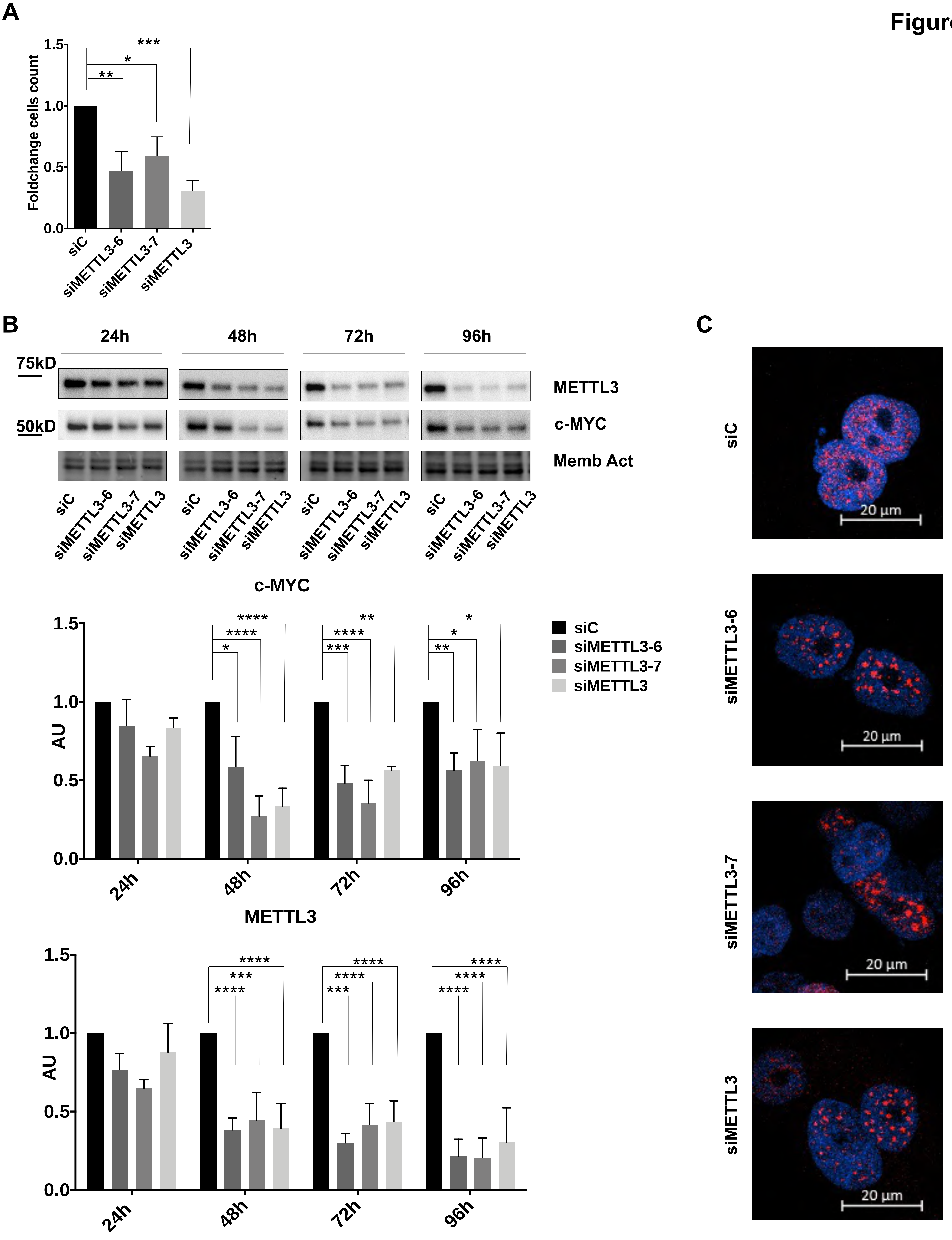

Suppl. Fig. 2. A TC1889 cells were transfected with siC, siMETTL3-6, siMETTL3-7 and siMETTL3 (a pool of four siRNA) and counted with Trypan blue at 96h (n=3). **B** Representative WB of c-MYC and METTL3 after 24, 48, 72 and 96h of METTL3-6, METTL3-7 and METTL3 depletion. Quantifications of three independent experiments are shown in the bottom panel. **C** Analysis of MALAT1 subcellular localization by RNA FISH in control (siC) and METTL3-silenced (siMETTL3-6, siMETTL3-7 and siMETTL3) samples at 72h.
