# Supplementary material for: METTL3-dependent MALAT1 delocalization drives c-Myc induction in thymic epithelial tumors
Source: Clin Epigenetics. 2021 Sep 16;13:173. doi: 10.1186/s13148-021-01159-6 (PMC8447796; doi:10.1186/s13148-021-01159-6)
Supplement: Supplementary file 3 — Additional file 3. Supplementary Figure 3. A) Puromycin and METTL3 quantification in the samples treated with Puromycin. The samples were normalized on membrane activation lanes (n = 4). B) Representative ribosomal fractionation profile after 72 h of control (siC, blue) and METTL3 silencing (siMETTL3, red) in TC1889 cells. On the right, calculation of area under the polysome and 80S curve in the siC and siMETTL3 samples at 72 h using ImageJ software (n = 2). C) Representative western blot of polysome after 48 h of METTL3 silencing. On the right c-MYC and METTL3 quantification, normalized on GAPDH (n = 3). [file 13148_2021_1159_MOESM3_ESM.pdf]

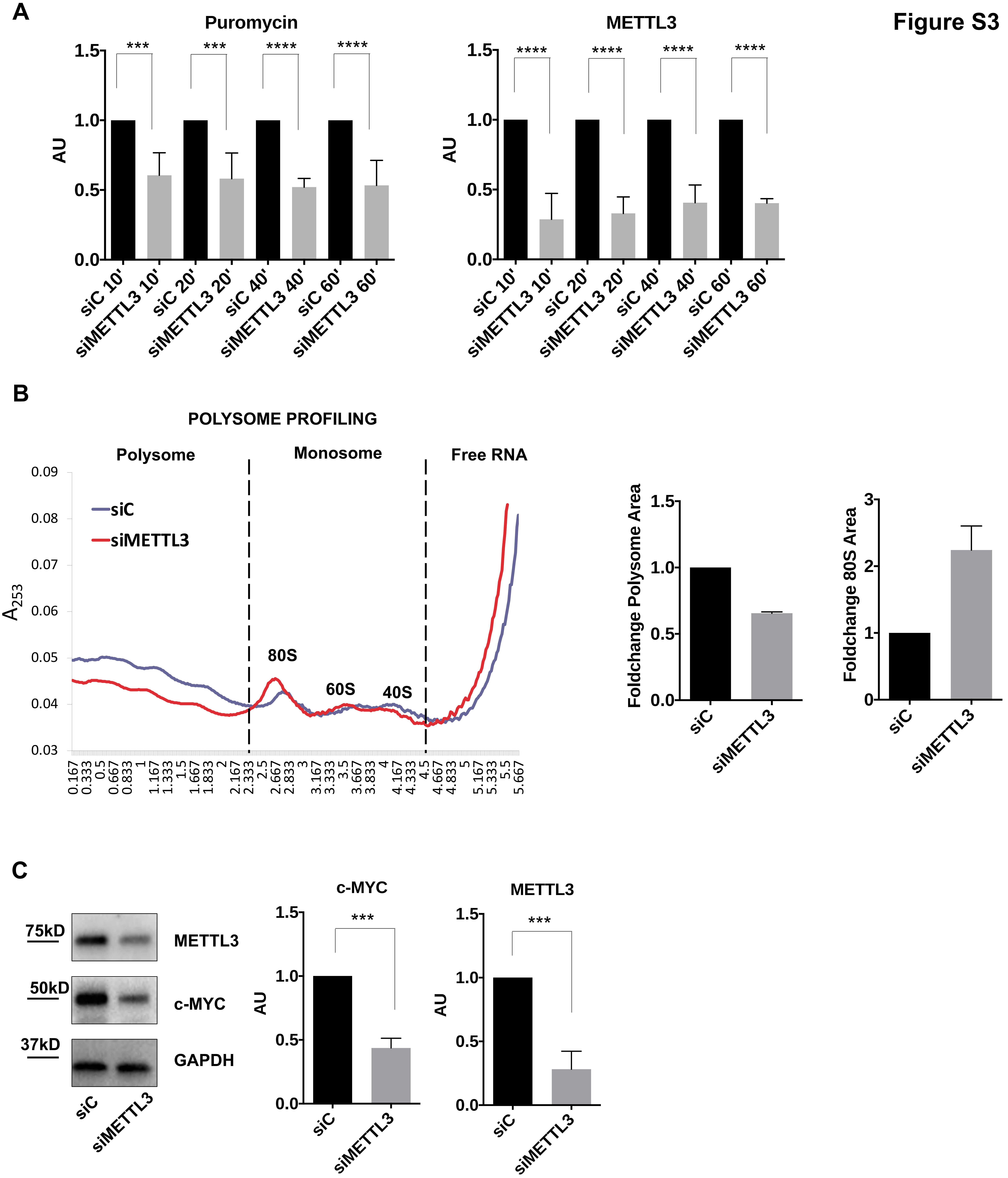

Suppl. Fig. 3. A Puromycin and METTL3 quantification in the samples treated with Puromycin. The samples were normalized on membrane activation lanes (n=4). **B** Representative ribosomal fractionation profile after 72h of control (siC, blue) and METTL3 silencing (siMETTL3, red) in TC1889 cells. On the right, calculation of area under the polysome and 80S curve in the siC and siMETTL3 samples at 72h using ImageJ software (n=2). **C** Representative western blot of polysome after 48h of METTL3 silencing. On the right c-MYC and METTL3 quantification, normalized on GAPDH (n=3).
