# Supplementary material for: METTL3-dependent MALAT1 delocalization drives c-Myc induction in thymic epithelial tumors
Source: Clin Epigenetics. 2021 Sep 16;13:173. doi: 10.1186/s13148-021-01159-6 (PMC8447796; doi:10.1186/s13148-021-01159-6)
Supplement: Supplementary file 4 — Additional file 4. Supplementary Figure 4. A) Malat1 expression after 72 h of silencing (n = 3). B) VEGF121 and VEGF165 expression was analyzed by qRT-PCR after 72 h of METTL3 and MALAT1 depletion in TET cells (n = 3). C) Representative Western blot of c-MYC after 24 h of METTL3 depletion (siC+siMETTL3 sample), 72 h of MALAT1 depletion (siC+siMALAT1 sample) and 24 h of METTL3 and 72 h of MALAT1 depletion (siMETTL3+siMALAT1 sample). On the top is represented c-MYC and METTL3 quantification, normalized on Membrane Activation (c-MYC n = 4, METTL3 n = 3), while on the bottom, c-MYC and MALAT1 expression was analyzed by qRT-PCR (n = 3). [file 13148_2021_1159_MOESM4_ESM.pdf]

Figure S4

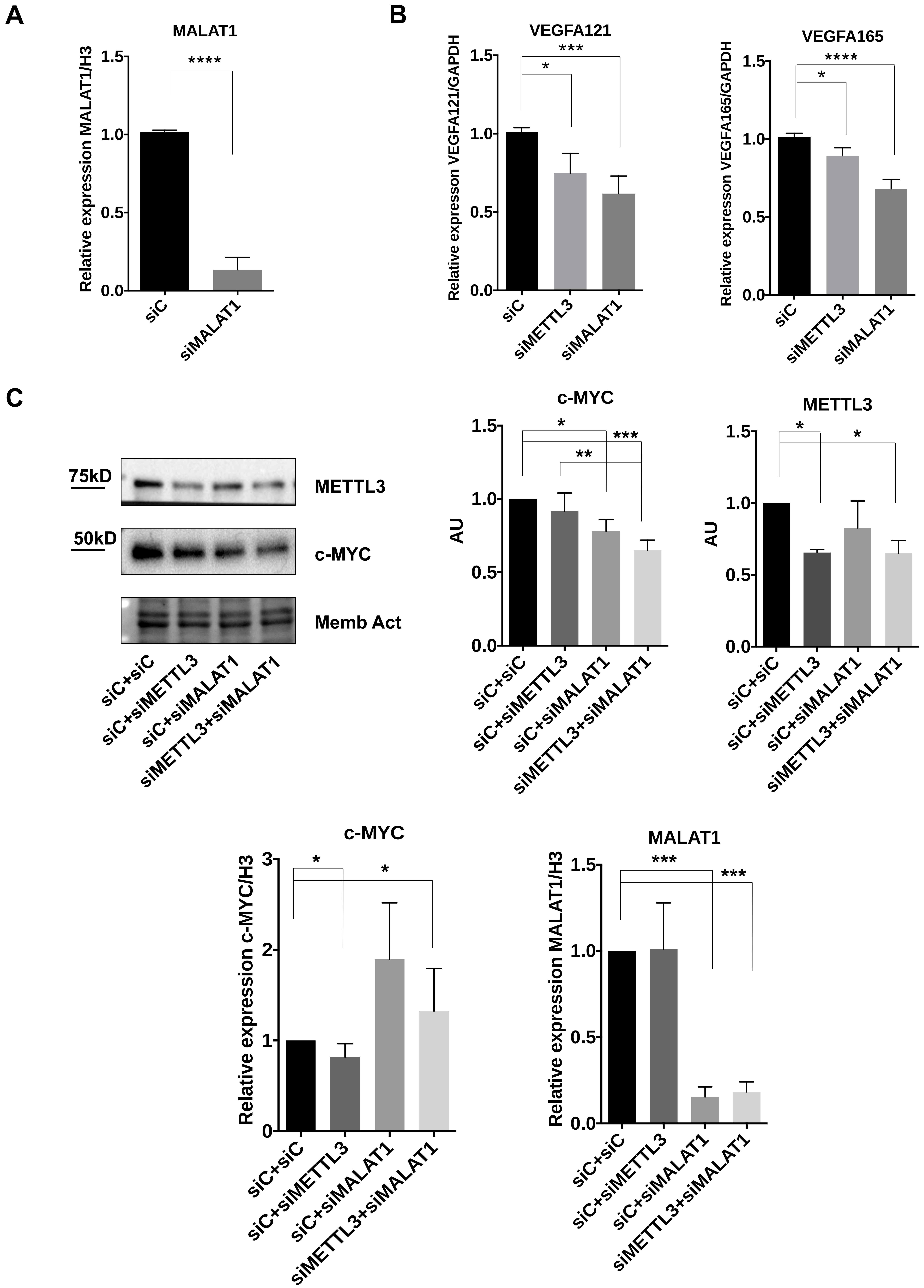

Suppl. Fig. 4. A Malat1 expression after 72h of silencing (n=3). **B** VEGF121 and VEGF165 expression was analyzed by qRT-PCR after 72h of METTL3 and MALAT1 depletion in TET cells (n=3). **C** Representative Western blot of c-MYC after 24h of METTL3 depletion (siC+siMETTL3 sample), 72h of MALAT1 depletion (siC+siMALAT1 sample) and 24h of METTL3 and 72h of MALAT1 depletion (siMETTL3+siMALAT1 sample). On the top is represented c-MYC and METTL3 quantification, normalized on Membrane Activation (c-MYC n=4, METTL3 n=3), while on the bottom, c-MYC and Malat1 expression was analyzed by qRT-PCR (n=3).
