# Supplementary material for: METTL3-dependent MALAT1 delocalization drives c-Myc induction in thymic epithelial tumors
Source: Clin Epigenetics. 2021 Sep 16;13:173. doi: 10.1186/s13148-021-01159-6 (PMC8447796; doi:10.1186/s13148-021-01159-6)
Supplement: Supplementary file 5 — Additional file 5. Supplementary Figure 5. A) METTL3 and c-MYC protein levels in TC cells treated with CDDP (top) and JQ1 (bottom) for 48 h. The treatment was performed 24 h after METTL3 silencing (n = 3). B) Representative Western Blot of Cleaved-PARP and PARP and on the right the ratio between Cleaved-PARP and PARP (n = 3). [file 13148_2021_1159_MOESM5_ESM.pdf]

Figure S5

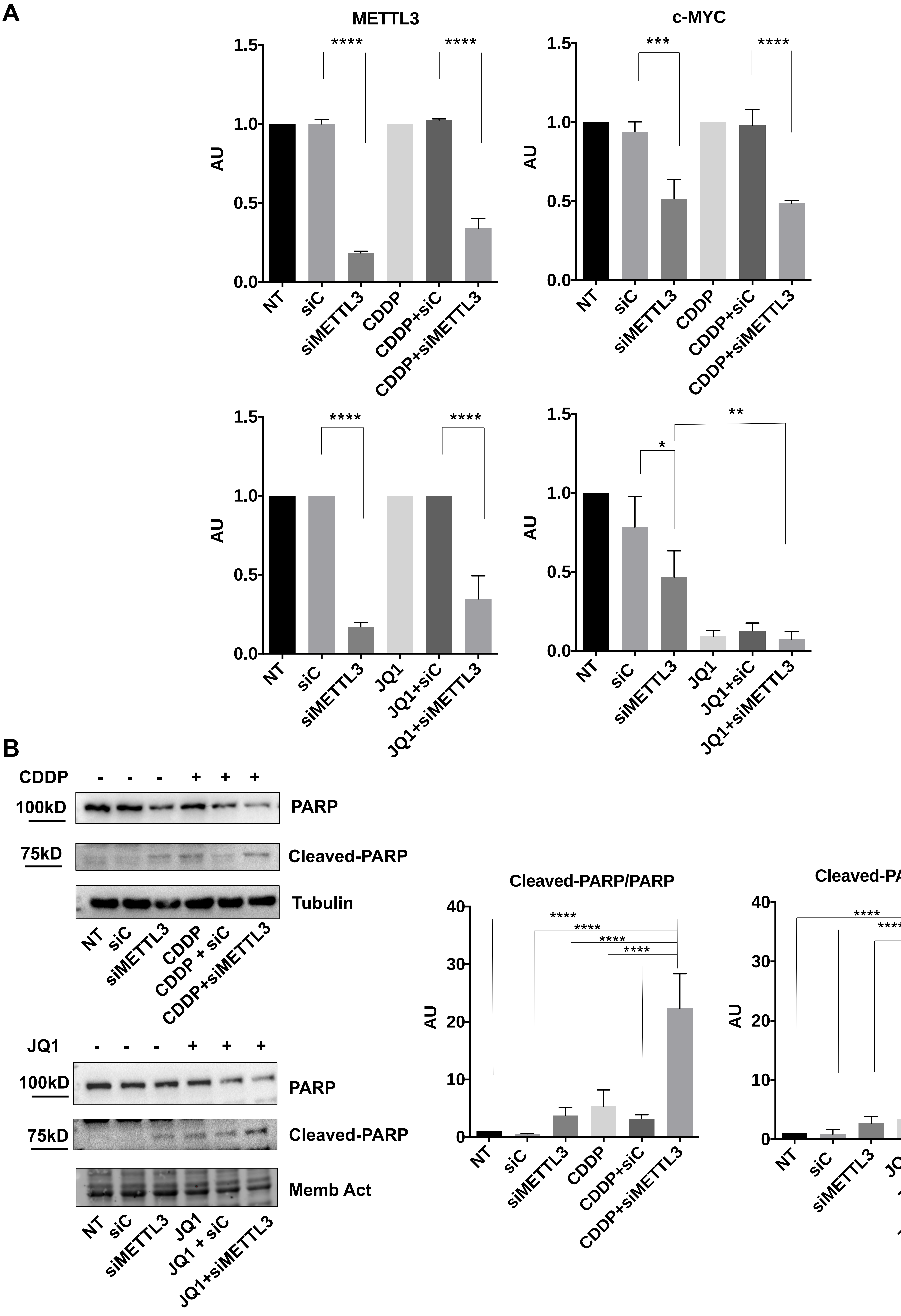

Suppl. Fig. 5. A METTL3 and c-MYC protein levels in TC cells treated with CDDP (top) and JQ1 (bottom) for 48h. The treatment was performed 24h after METTL3 silencing (n=3). **B** Representative Western Blot of Cleaved-PARP and PARP and on the right the ratio between Cleaved-PARP and PARP (n=3).
